# Factors Associated with Exclusive Breast-feeding in Japan: for Activities to Support Child-rearing with Breast-feeding

**DOI:** 10.2188/jea.16.57

**Published:** 2006-03-14

**Authors:** Akiyo Kaneko, Yoshitaka Kaneita, Eise Yokoyama, Takeo Miyake, Satoru Harano, Kenshu Suzuki, Eiji Ibuka, Takako Tsutsui, Yuko Yamamoto, Takashi Ohida

**Affiliations:** 1 Department of Public Health, School of Medicine, Nihon University.

**Keywords:** Breast Feeding, Logistic Models, Multiple Birth Offspring, Smoking, Japan

## Abstract

**BACKGROUND:**

Benefits of breast-feeding are not only limited to nutrition and sanitation in developing countries but also extend to cost-saving health care and alleviation of anxiety related to childrearing in developed countries. This study aims to elucidate factors associated with exclusive breast-feeding in Japan and use this information to achieve child-rearing support worldwide by promoting breast-feeding.

**METHODS:**

This cross-sectional study used data from a survey conducted by Ministry of Health, Labour and Welfare of the Japanese government, the First Longitudinal Survey of Babies in 21st Century. All subjects were infants (n = 53,575) born in Japan in 2001 between January 10 and 17 and between July 10 and 17. According to the data, the exclusive breast-feeding rate in Japan during the first 6 months of life was 21.0%. We examined the factors associated with exclusive breast-feeding using univariate and multivariate logistic regression analyses.

**RESULTS:**

Among the factors examined, the adjusted odds ratio (OR) for exclusive breast-feeding was low for late childbearing, low birth weight infants, multiple births, smoking parents, living with grandparents, and feeling burdened by childrearing. The adjusted OR was high for factors that included sufficient childcare leave and consultation about childrearing with the spouse, a birth attendant and/or nurse, and a peer in a child-rearing circle.

**CONCLUSIONS:**

Exclusive breast-feeding is associated not only with medical factors but also with social factors. This study clarifies the necessity of social support to reduce the child rearing burden and a political system to promote paternal participation in childrearing and to improve the childcare leave system.

While infant formulas have penetrated markets worldwide through the marketing strategies of formula manufacturers and other factors beginning in the 1950s and extending through the 1960s, the breast-feeding rate has declined. This decline has resulted in an increased infant mortality rate due to impaired immunity, particularly in developing countries.^1^ In order to address this serious problem, the World Health Organization (WHO), the United Nations Children’s Fund (UNICEF), and other organizations have endeavored to disseminate information on the superiority of breast-feeding and increase the breast-feeding rate worldwide. For example, in 1980, the WHO formulated the “International Code of Marketing of Breast-milk Substitutes,” commonly known as the WHO code, and warned manufacturers against engaging in aggressive sales promotion of substitutes for breast-feeding, such as formula feeding, to mothers, irrespective of the actual need. Furthermore, the “Global Strategy for Infant and Young Child Feeding” adopted by the WHO and the UNICEF in 2001 recommended exclusive breast-feeding, that is, providing only breast milk and not using any infant formulas during the first 6 months of life.^2^

In the past, the exclusive breast-feeding rate in Japan during the first month of an infant’s life was more than 70%. However, consequent to the spread of formula feeding from the 1960s through the 1970s, that rate fell to 31.7%.^3^^-^^4^ Following this decline, the advantages of breast-feeding were reevaluated; however, the most recent data on the exclusive breast-feeding rate during the first month of life shows that it has recovered to only approximately 45%.^3^^-^^4^

Although breast-feeding is an important irreplaceable act for both mothers and children that provides maternal health benefits, builds the relationship between the mother and the child, and promotes the health of the child, why do many mothers abandon it? Undoubtedly, there exist conditions that require formula feeding, such as premature delivery, low birth weight, or the prevention of infection through breast-feeding. However, it appears that formula feeding is chosen because of lack of knowledge about the benefits of breast-feeding and social reasons such as conditions at the workplace. According to the First Longitudinal Survey of Babies in 21st Century conducted in 2001, the exclusive breast-feeding rate and the rate of using a combination of breast-feeding and formula feeding in Japan was 21.0% and 72.7%, respectively, during the first 6 months of life.^5^ However, the factors associated with exclusive breast-feeding have not been sufficiently examined.

Therefore, this study aims to elucidate the types of factors associated with exclusive breast-feeding using data from the First Longitudinal Survey of Babies in 21st Century. The recovery of the breast-feeding rate to its former level is considered to be extremely difficult to achieve. We hope that the results of this study will be useful in formulating tactics to support breast-feeding in developed countries such as Japan as well as in developing countries.

## METHODS

### Data Collection

This study used data from the First Longitudinal Survey of Babies in 21st Century conducted by the Ministry of Health, Labour and Welfare of the Japanese government and from the vital statistics data (*jinko dotai chosa shusseihyo*).^6^ All subjects were infants born in Japan in 2001 between January 10 and 17 and between July 10 and 17. The total number of subjects born in the surveyed period was 53,575 (infants born in January: 26,620; infants born in July: 26,955). This number corresponds to 4.6% of the infants born in Japan in 2001. The survey was conducted when the infants were 6 months of age. For this survey, the Ministry of Health, Labour and Welfare mailed a self-administered questionnaire to the subjects’ households so that a family member could complete the questionnaire and return it by mail. The questionnaire included the following items: the number of family members living together; maternal employment status; current parental smoking status; the feeling of being burdened by childrearing; and anxieties and worries related to childrearing, feeding, and the status of the household’s annual income. Three alternatives were provided for breast-feeding (breast-fed, fed only colostrum, or did not breast-feed) and two alternatives were provided for formula feeding (fed or not fed on it). Those who fed breast milk or formula milk were required to specify the duration of each feeding modality. Additionally, through the vital statistics data based on birth certificates, the Ministry collected data on the mother’s date of birth, place of residence, infant’s date of birth, infant’s birth weight, infant’s gestational age (weeks), and whether the mother had multiple births. This data was linked to the data obtained from the returned questionnaire, thereby creating a data form for each infant.

We used the data that had already linked the First Longitudinal Survey of Babies in 21st Century to the vital statistics data after seeking permission from the Ministry of Health, Labour and Welfare. Before granting permission, the Ministry deleted all identifiers till that date on the database in order to protect the privacy of subject infants and their families.

### Data Analysis

The total number of collected questionnaires was 47,010 (infants born in January: 23,421; infants born in July: 23,589) with a collection rate of 87.7% (infants born in January: 88.0%; infants born in July: 87.5%). Infants who were not living with their mothers were excluded from this study (n = 49). Further, the infants for whom questions related to feeding were not answered were also excluded (n = 392), leaving a total of 46,569 infants for analysis.

We calculated the exclusive breast-feeding rate according to each possible factor (Table 1). We examined demographic characteristics (maternal age, the number of deliveries by the mother, the place of residence, if the mother was living with the father or with grandparents, annual family income, and maternal employment status), infants’ characteristics (sex, birth weight, gestational age in weeks, multiple or single births, and the month of birth), the current smoking status of parents, and factors related to childrearing (childcare advisors and the feeling of being burdened by childrearing).

**Table 1.** Prevalence of exclusive breast-feeding, univariate and multivariate odds ratios (ORs) and their 95% confidence intervals (CIs) for factors associated with exclusive breast-feeding during the first 6 months of life in Japan.

	Total number (n)	prevalence of exclusive breast-feeding (%)	Univariate	Multivariate
	
			Crude OR (95% CI)	Adjusted OR 95% CI)
	***Demographic characteristics***			
Maternal age (year, n=46.569)				
-19	173	12.1	0.57 (0.36-0.91)	0.67 (0.37-1.24)
20-29	17353	21.0	1.00 (reference)	1.00 (reference)
30-39	27583	22.4	1.20 (1.14-1.26)	0.89 (0.84-0.94)
40-	1460	16.3	0.81 (0.70-0.94)	0.56 (0.48-0.65)

No. of deliveries by mother (n=46.569)				
first	23286	17.2	1.00 (reference)	1.00 (reference)
second	17121	24.5	1.57 (1.49-1.65)	1.72 (1.63-1.81)
third+	6324	26.1	1.71 (1.60-1.82)	2.06 (1.91-2.22)

Place of residence (n=46.596)				
urban	9976	21.9	1.07 (1.01-1.13)	1.07 (1.01-1.13)
suburban	27686	20.8	1.00 (reference)	1.00 (reference)
rural	8907	20.9	1.01 (0.98-1.07)	1.03 (0.97-1.10)

Living with father (n=46.569)				
Yes	45525	21.2	1.00 (reference)	1.00 (reference)
No	1044	13.8	0.59 (0.50-0.71)	0.83 (0.60-1.17)

Living with grandparent (n=46.569)				
Yes	100296	18.6	1.00 (reference)	1.00 (reference)
No	36276	21.7	1.21 (1.15-1.28)	1.14 (1.07-1.21)

Annual family income (million yen, n=46.569)				
-3.9	11837	19.0	0.85 (0.80-0.90)	0.93 (0.88-0.99)
4.0-7.9	24652	21.6	1.00 (reference)	1.00 (reference)
8.0-	10080	22.0	1.03 (0.97-1.08)	1.03 (0.97-1.09)

Maternal employment status (n=45.925)				
non working	34240	22.2	1.00 (reference)	1.00 (reference)
full-time employee				
(childcare leave 6+ mo)	4697	25.0	1.17 (1.09-1.26)	1.14 (1.05-1.23)
(childcare leave <6 mo)	1082	6.7	0.25 (0.20-0.32)	0.26 (0.21-0.34)
(without childcare leave)	1389	4.2	0.17 (0.12-0.20)	0.16 (0.12-0.21)
part-timer	1945	12.0	0.48 (0.42-0.55)	0.49 (0.42-0.57)
self-supporting work	1994	21.8	0.98 (0.88-1.09)	0.93 (0.83-1.04)
side job	481	20.2	0.89 (0.71-1.11)	0.84 (0.67-1.06)
student	97	14.4	0.59 (0.34-1.05)	0.72 (0.39-1.30)

	***Infant’s characteristics***			
Infant sex (n=46.569)				
male	24222	20.6	1.00 (reference)	1.00 (reference)
female	22347	21.5	1.06 (1.01-1.11)	1.06 (1.01-1.11)

Birth weight (kg, n=46.557)				
-2499	3960	11.4	0.46 (0.42-0.51)	0.67 (0.60-0.76)
2500-	42597	21.9	1.00 (reference)	1.00 (reference)

Gestational ages weeks (n=46.157)				
-36	2394	10.7	0.43 (0.38-0.49)	0.66 (0.57-0.76)
37-	43763	21.6	1.00 (reference)	1.00 (reference)

single birth, multiple births (n=46.569)				
single birth	45594	21.5	1.00 (reference)	1.00 (reference)
multiple births	975	1.3	0.05 (0.03-0.09)	0.07 (0.04-0.12)

Month of birth (n=46.569)				
July	23448	21.7	1.00 (reference)	1.00 (reference)
January	23121	20.4	0.93 (0.89-0.97)	0.94 (0.90-0.99)

Current smoking status of the mother and father (n=46.324)				
Nil	16166	24.5	1.00 (reference)	1.00 (reference)
only father	21819	22.6	0.90 (0.86-0.95)	0.92 (0.88-0.97)
only mother	579	11.5	0.40 (0.31-0.95)	0.44 (0.34-0.57)
father and mother	7236	10.3	0.36 (0.33-0.39)	0.37 (0.34-0.40)

	***Factors related to child-rearing***			
Advisors on childcare				
Spouse (n=46.569)				
Yes	38033	21.8	1.28 (1.21-1.37)	1.07 (1.00-1.14)
No	8536	17.8	1.00 (reference)	1.00 (reference)

Parents of mother (n=46.569)				
Yes	33736	21.1	1.01 (0.96-1.06)	1.00 (0.94-1.05)
No	12833	21.0	1.00 (reference)	1.00 (reference)

Parents of father (n=46.569)				
Yes	14122	21.8	1.06 (1.01-1.12)	1.01 (0.96-1.07)
No	32447	20.7	1.00 (reference)	1.00 (reference)

Birth attendant/nurse (n=46.569)				
Yes	2499	29.7	1.63 (1.50-1.78)	1.76 (1.60-1.94)
No	42991	20.5	1.00 (reference)	1.00 (reference)

Childcare professionals (n=46.569)				
Yes	2550	18.9	0.87 (0.78-0.97)	0.95 (0.84-1.07)
No	44310	21.1	1.00 (reference)	1.00 (reference)

Peer in child rearing circle (n=46.569)				
Yes	2807	27.6	1.47 (1.35-1.60)	1.25 (1.14-1.37)
No	43762	20.6	1.00 (reference)	1.00 (reference)

Do you have any problems regarding childcare? (n=46.296)				
Yes	37024	20.3	0.81 (0.77-0.85)	0.81 (0.76-0.86)
No	46296	24.0	1.00 (reference)	1.00 (reference)

All variables in the table are used as independent variables for logistic analysis.

Cases for which the value was unknown for a variable were excluded in that variable’s analysis.

Logistic regression analysis was used to determine the factors associated with exclusive breast-feeding. Univariate and multivariate logistic regression analyses were performed using exclusive breast-feeding during the first 6 months of life as the dependent variable and each associated factor as the independent variable.

Statistical analysis employed the use of SPSS^®^ 11.0J for Windows. Two-sided p values equal to or less than 0.05 were considered to be statistically significant.

## RESULTS

Table 1 shows the results of logistic regression analysis.

With regard to maternal age, univariate analysis showed that the crude odds ratio (OR) was highest for women in their 30s and multivariate analysis showed that the adjusted OR was highest for women in their 20s. Multivariate analysis showed that being older than 30 was a significant negative factor associated with exclusive breast-feeding. The adjusted OR for exclusive breast-feeding increased with the number of deliveries.

Multivariate analysis showed that living with the father was not associated with exclusive breast-feeding. On the other hand, the adjusted OR was significantly lower for women living with their grandparents than for those not living with them, suggesting that living with grandparents was a negative factor associated with exclusive breast-feeding.

With regard to maternal employment status, the adjusted OR of taking sufficient childcare leave (6 months or longer) even when the mother was employed in a full-time job was 1.14 (95% confidence interval [CI]: 1.05–1.23). When compared with being unemployed, sufficient childcare leave was found to be a positive factor associated with exclusive breast-feeding. Mothers who took insufficient childcare leave (less than 6 months), those who did not take childcare leave, and those who were employed as part-time workers were found to be negatively associated with exclusive breast-feeding.

Low birth weight, premature birth, and multiple births were negatively associated with exclusive breast-feeding. In the case of multiple births, the adjusted OR was found to be significantly lower at 0.07 (95% CI: 0.04–0.12), which was a strong negative factor for exclusive breast-feeding.

When compared with nonsmoking parents, the adjusted OR was significantly lower for all those with a smoking status (only father is a smoker, only mother is a smoker, or both parents are smokers), suggesting that smoking by parents as well as mothers was negatively associated with exclusive breast-feeding.

With regard to childcare advisors, univariate and multivariate analyses showed that consultation about childbearing with the spouse, a birth attendant/nurse, or a peer in a child-rearing circle was a positively associated factor for exclusive breast-feeding. The adjusted OR for feeling burdened by childrearing was compared with that of those who did not have such a feeling and was found to be 0.81 (95% CI: 0.76–0.86); this feeling was a negative factor for exclusive breast-feeding.

This analysis included both single birth and multiple births. In order to eliminate the influence of multiple births, we performed a supplemental analysis on single birth, which is the same as the analysis shown in Table 1. The results of the supplemental analysis did not differ from those for the overall subject group. Multiple births did not affect the validity of the overall results.

## DISCUSSION

The First Longitudinal Survey of Babies in 21st Century — from which data were used in this study — is a large scale survey with a sample size of more than 40,000 selected from all the regions of Japan. This survey was conducted by the Ministry of Health, Labour and Welfare. The collection rate was extremely high, and sampling was performed in order to represent the situation of 6 months old infants throughout Japan. Therefore, the above data appears to be very useful for planning measures to improve health care for mothers and infants in Japan.

According to the data from this survey, the colostrum feeding rate in Japan was 98.3%,^5^ which was extremely high as compared with reports from other developed countries (Table 2).^7^^-^^11^ Colostrum — the breast milk secreted for the first 4 or 5 days postpartum — is rich in immunoglobulin A and white blood cells and is considered to play an important role in the infant’s immune system.^12^ The colostrum feeding rate was found to be particularly high because its benefits are well known to not only medical professionals but also mothers. On the other hand, the exclusive breast-feeding rate could not be compared with that in other countries because the feeding modality and the duration surveyed varied in each country. Exclusive breast-feeding during the first 6 months of life is recommended by the WHO/UNICEF. Therefore, it appears to be an appropriate marker for international comparison of breast-feeding patterns. We await reports from other developed countries regarding this situation.

**Table 2.** Prevalence of colostrum feeding in selected developed countries and areas.

Country	year	n	colostrum feeding (%)
Japan	2001	46569	98.3 (98.2-98.4)
Italy^7^	1999	2771	91.1 (90.0-92.2)
New Zealand^8^	1994	1592	85.0 (83.2-86.8)
U.K.^9^	2000	720	69.0 (65.6-72.4)
U.S.A.^10^	1998	96204	67.5 (67.2-67.8)
Hong Kong^11^	1997	7825	33.5 (32.5-34.5)

95% confidence intervals in parentheses

With regard to maternal age, the crude OR for exclusive breastfeeding was highest for women in their 30s of age; however, the adjusted OR, as determined by multivariate analysis, showed the rate to be highest for women in their 20s. With regard to the explanation for the confounding factors that could increase the exclusive breast-feeding rate in women in their 30s, as observed by univariate analysis, it was suggested that women in their 30s have more deliveries and are less likely to be smokers than the women in their 20s. These data showed that the smoking rate among women in their 20s (25.2%) was significantly higher than that among those in their 30s (12.7%).

We predicted that living with grandparents, who were experienced in childrearing, would be associated with exclusive breastfeeding. However, contrary to the prediction, this living situation significantly decreased the OR for exclusive breast-feeding. In the 1960s and the 1970s, when the majority of the parents of the subject infants were born, the breast-feeding rate in Japan had reached a nadir. The grandparents of the infants for whom this survey was intended belonged to the generation that had fed formulas to their own children. For this reason, living with grandparents might be associated with reluctance to breast-feeding on the basis of the information provided by the grandparents on the merits of formula feeding.

The adjusted OR for exclusive breast-feeding was higher for mothers who were employed in full-time jobs and yet were able to take sufficient childcare leave than it was for unemployed mothers. This reconfirmed the importance of the childcare leave system. According to the Child Care and Family Care Leave Act implemented in 1992, workers have the right to take childcare leave until their child reaches the age of one year. Unemployment insurance provides payment to parents during this leave period, which is approximately 40% of the monthly wage beginning at the time of the leave. However, because the guaranteed wage during childcare leave is not sufficient and the development of a working environment that supports childrearing has been delayed, the utilization rate of childcare leave is 0.33% for males and 64.0% for females.^13^

It was found that low birth weight and premature birth did not necessarily have the same implication; however, when analyzed at the same time, they could possibly lead to the problem of collinearity. In fact, two methods of analysis were used in the preliminary study: the first method involved the analysis of low birth weight,^10^^,^^11^ and the second method involved the simultaneous analysis of both low birth weight and premature birth.^7^^-^^9^ We used three methods of multivariate analysis (only low birth weight, only premature birth, and both low birth weight and premature birth) to examine whether the problem of collinearity existed between low birth weight and premature birth; however, the data indicating the existence of the problem of collinearity were not recognized.

The multiple births factor was found to have the lowest adjusted OR in this study. There is no doubt that multiple births can cause not only medical complications but also various other problems related to family structure, economic status, and childcare. Assisted reproductive technology has recently made remarkable advances, resulting in a rapid increase of the number of multiple births.^14^ Therefore, prompt resolution of these problems has become a matter of high priority.

With regard to the current maternal smoking status, the possible reasons why smoking mothers tended not to exclusively breast-feed their children would be that they were concerned about transferring nicotine through breast milk or that they had difficulty secreting breast milk due to increased somatostatin levels in the blood as compared with nonsmoking mothers.^15^^-^^17^ According to a report by Ohida et al., the smoking rate among young women is high in Japan; the smoking rate among women in their 20s is 28.8%, of which, 10.9% continue to smoke even during pregnancy.^18^ In this study, not only maternal smoking but also paternal smoking was found to be a negatively associated factor for exclusive breast-feeding. The possibility of paternal smoking suppressing the initiation of colostrum feeding by the mother was reported previously,^15^ and the result supports our study. With regard to the relationship between paternal smoking and maternal breast-feeding, it can be assumed that the mother is affected by the passive smoke caused by the father’s smoking habit, which is turn affects the mother’s attitude toward childcare. Regardless of sex, a smoking cessation program targeting this generation is an urgent requirement from the viewpoint of breast-feeding.

The factor that was positively associated with exclusive breast-feeding was childcare advisors: consultation with the spouse, a birth attendant/nurse, or a peer in a child-rearing circle. It was likely that the relationship between mothers and fathers and the fact that they discussed childcare had a desirable influence on breast-feeding. With regard to recent health care initiative establishing the importance of paternal childcare participation, the results of this study provide useful information. Consultation with a birth attendant/nurse was a positive factor associated with exclusive breast-feeding because they guide and recommend exclusive breast-feeding in a positive manner. Further, the child-rearing circle appeared to play an important role as a reliable source of information and support for mothers.

This study has several limitations. First, this survey utilized a self-administered questionnaire, which was filled out by a family member of the subject. Although confidentiality was completely guaranteed, mothers may have possibly felt a sense of guilt with regard to childrearing without breast-feeding, which may have resulted in false reports and an overestimation of the rate of breast-feeding. Second, because this study is cross-sectional, causal factors cannot be inferred. Third, only three situations were assumed: (1) the mothers had no intention to exclusively breast-feed during the first 6 months of life, (2) they had the intention but could not secrete breast milk, and (3) they had the intention to breast-feed and could secrete breast milk, but could not breast feed for certain other reasons. The questionnaire used for this study did not collect the data that could differentiate among the three situations. Fourth, this survey did not include questions that are included in the surveys conducted by other countries, such as “method of delivery,”^8^^,^^9^ “presence or absence of treatment in a neonatal intensive care unit,”^7^^,^^8^ “maternal education,”^9^^,^^10^ and “drinking status of the mother,”^8^ as well as other questions that would prove useful in further evaluations such as “the presence or absence of pregnancy complications”. These points will be the themes for a future study.

There is an opinion that the guidelines promoted by the WHO/UNICEF cannot be applied to the current situation in developed countries, including Japan. Nevertheless, when viewed from different perspectives, the value of breast-feeding in developed countries is equal to or greater than that in developing countries. Reports show that breast-fed infants are not only less likely to contract infectious diseases^19^^,^^20^ but also are less likely to be overweight^21^^,^^22^ and more likely to have a lower incidence rate of allergic disease.^23^ Further, breast-feeding has been reported to establish a better relationship between the mother and the child, which leads to a reduction in the number of abused children.^24^^,^^25^ Furthermore, the development of a social environment that allows a mother to easily choose breast-feeding for her child could reduce the mother’s anxiety with regard to childrearing and ensure full participation of women in society. The WHO/UNICEF has prioritized the promotion of breast-feeding worldwide and has concluded that the appropriate period for exclusive breast-feeding is the first 6 months of life. However, the most appropriate period of breast-feeding has been the focus of recent epidemiologic studies on breast-feeding.

Late childbearing, low birth weight infants, and multiple births were found to be the negative factors associated with exclusive breast-feeding; therefore, breast-feeding support should be prioritized for mothers who belong to these groups. Health policies within the country, such as the childcare leave system, and childrearing support in the community, such as child-rearing circles, and paternal childcare participation were suggested to be essential for exclusive breast-feeding. Recently, the national campaign “Sukoyaka (healthy and happy) Family 21” promoted by the Ministry of Health, Labour and Welfare of the Japanese government has established the increase in the breast-feeding rate as one of it objectives. In addition, “Basic Direction for Future Child Rearing Support Measures,” also known as the “New Angel Plan,” has been devised by the Ministry of Health, Labour and Welfare to develop an anti-declining birth rate measure to be carried out over a period of 10 years starting from 1995; in other words, it supports voluntary activities in the community, such as child-rearing circles. Further development of this type of social support is critical for the promotion of breast-feeding.

In order to promote breast-feeding, more effective measures tailored to various conditions related to culture, the social situation, and the level of health care in each country are required. We hope that this report from Japan will help the cause of increasing breast-feeding in Japan and in other countries.
