# Vol.16 (2006), No.2 pp.57

**DOI:** 10.2188/jea.16.136

**Published:** 2006-05-19

RE: “Factors Associated with Exclusive Breast-feeding in Japan: for Activities to Support Child-rearing with Breast-feeding”

About the paper by Kaneko A et al. published in the March 2006 issue of the *Journal*,^1^ the authors requested that some errors in the Table 1 (p59) of the original paper should be corrected. The revised version of the Table is on the next page.

**Table 1.** Prevalence of exclusive breast-feeding, univariate and multivariate odds ratios (ORs) and their 95% confidence intervals (CIs) for factors associated with exclusive breast-feeding during the first 6 months of life in Japan.

	Total number (n)	prevalence of exclusive breast-feeding (%)	Univariate	Multivariate
			Crude OR (95% CI)	Adjusted OR (95% CI)
	***Demographic characteristics***			
Maternal age (year, n=46,569)				
-19	173	12.1	0.57 (0.36-0.91)	0.67 (0.37-1.24)
20-29	17353	19.4	1.00 (reference)	1.00 (reference)
30-39	27583	22.4	1.20 (1.14-1.26)	0.89 (0.84-0.94)
40-	1460	16.3	0.81 (0.70-0.94)	0.56 (0.48-0.65)

No. of deliveries by mother (n=46,569)				
first	23286	17.2	1.00 (reference)	1.00 (reference)
second	16962	24.5	1.56 (1.49-1.64)	1.72 (1.63-1.81)
third+	6321	26.1	1.71 (1.60-1.82)	2.06 (1.91-2.22)

Place of residence (n=46,569)				
urban	9976	21.9	1.07 (1.01-1.13)	1.07 (1.01-1.14)
suburban	27686	20.8	1.00 (reference)	1.00 (reference)
rural	8907	20.9	1.01 (0.95-1.07)	1.03 (0.97-1.10)

Living with father (n=46,569)				
Yes	45525	21.2	1.00 (reference)	1.00 (reference)
No	1044	13.8	0.59 (0.50-0.71)	0.83 (0.60-1.17)

Living with grandparent (n=46,569)				
Yes	10296	18.6	1.00 (reference)	1.00 (reference)
No	36273	21.7	1.21 (1.15-1.28)	1.14 (1.07-1.21)

Annual family income (million yen, n=46,569)				
-3.9	11837	19.0	0.85 (0.80-0.90)	0.93 (0.88-0.99)
4.0-7.9	24652	21.6	1.00 (reference)	1.00 (reference)
8.0-	10080	22.0	1.03 (0.97-1.08)	1.03 (0.97-1.09)

Maternal employment status (n=45,925)				
non working	34240	22.2	1.00 (reference)	1.00 (reference)
full-time employee				
(childcare leave 6+ mo)	4697	25.0	1.17 (1.09-1.26)	1.14 (1.05-1.23)
(childcare leave <6 mo)	1082	6.7	0.25 (0.20-0.32)	0.26 (0.21-0.34)
(without childcare leave)	1389	4.2	0.16 (0.12-0.20)	0.16 (0.12-0.21)
part-timer	1945	12.0	0.48 (0.42-0.55)	0.49 (0.42-0.57)
self-supporting work	1994	21.8	0.98 (0.88-1.09)	0.93 (0.83-1.04)
side job	481	20.2	0.89 (0.71-1.11)	0.84 (0.67-1.06)
student	97	14.4	0.59 (0.34-1.05)	0.72 (0.39-1.30)

	***Infant’s characteristics***			
Infant sex (n=46,569)				
male	24222	20.6	1.00 (reference)	1.00 (reference)
female	22347	21.5	1.06 (1.01-1.11)	1.06 (1.01-1.11)

Birth weight (g, n=46,557)				
-2499	3960	11.4	0.46 (0.42-0.51)	0.67 (0.60-0.76)
2500-	42597	21.9	1.00 (reference)	1.00 (reference)

Gestational ages weeks (n=46,157)				
-36	2394	10.7	0.43 (0.38-0.49)	0.66 (0.57-0.76)
37-	43763	21.6	1.00 (reference)	1.00 (reference)

single birth, multiple births (n=46,569)				
single birth	45594	21.5	1.00 (reference)	1.00 (reference)
multiple births	975	1.3	0.05 (0.03-0.09)	0.07 (0.04-0.12)

Month of birth (n=46,569)				
July	23448	21.7	1.00 (reference)	1.00 (reference)
January	23121	20.4	0.93 (0.89-0.97)	0.94 (0.90-0.99)

	***Current smoking status of the mother and father***			
Smoking status (n=45,430)				
Nil	16058	24.5	1.00 (reference)	1.00 (reference)
only father	21630	22.6	0.90 (0.86-0.95)	0.92 (0.88-0.97)
only mother	572	11.5	0.40 (0.31-0.52)	0.44 (0.34-0.57)
father and mother	7170	10.3	0.36 (0.33-0.39)	0.37 (0.34-0.40)

	***Factors related to child-rearing***			
Advisors on childcare				
Spouse (n=46,569)				
Yes	38033	21.8	1.28 (1.21-1.37)	1.07 (1.00-1.14)
No	8536	17.8	1.00 (reference)	1.00 (reference)

Parents of mother (n=46,569)				
Yes	33736	21.1	1.01 (0.96-1.06)	1.00 (0.94-1.05)
No	12833	21.0	1.00 (reference)	1.00 (reference)

Parents of father (n=46,569)				
Yes	14122	21.8	1.06 (1.01-1.12)	1.01 (0.96-1.07)
No	32447	20.7	1.00 (reference)	1.00 (reference)

Birth attendant/nurse (n=46,569)				
Yes	2550	29.7	1.63 (1.50-1.78)	1.76 (1.60-1.94)
No	44019	20.5	1.00 (reference)	1.00 (reference)

Childcare professionals (n=46,569)				
Yes	2259	18.9	0.87 (0.78-0.97)	0.95 (0.84-1.07)
No	44310	21.1	1.00 (reference)	1.00 (reference)

Peer in child rearing circle (n=46,569)				
Yes	2807	27.6	1.47 (1.35-1.60)	1.25 (1.14-1.37)
No	43762	20.6	1.00 (reference)	1.00 (reference)

Do you have any problems regarding childcare? (n=46,296)				
Yes	37024	20.3	0.81 (0.77-0.85)	0.81 (0.76-0.86)
No	9272	24.0	1.00 (reference)	1.00 (reference)

All variables in the table are used as independent variables for logistic analysis.

Cases for which the value was unknown for a variable were excluded in that variable’s analysis.

The *Journal* regrets the errors.
